# Fisetin Inhibits Osteogenic Differentiation of Mesenchymal Stem Cells via the Inhibition of YAP

**DOI:** 10.3390/antiox10060879

**Published:** 2021-05-30

**Authors:** Chanchao Lorthongpanich, Thanapon Charoenwongpaiboon, Prapasri Supakun, Methus Klaewkla, Pakpoom Kheolamai, Surapol Issaragrisil

**Affiliations:** 1 Siriraj Center of Excellence for Stem Cell Research, Department of Medicine, Faculty of Medicine Siriraj Hospital, Mahidol University, Bangkok 10700, Thailand; cup14t@gmail.com (P.S.); surapolsi@gmail.com (S.I.); 2 Department of Chemistry, Faculty of Science, Silpakorn University, Nakhon Pathom 73000, Thailand; charoenwongpaib_t@su.ac.th; 3 Department of Biochemistry, Faculty of Science, Chulalongkorn University, Bangkok 10330, Thailand; methus.kanon@gmail.com; 4 Division of Cell Biology, Faculty of Medicine, Thammasat University, Pathum Thani 10120, Thailand; pkheolamai@me.com

**Keywords:** fisetin, osteogenic differentiation, mesenchymal stem cells, inhibition, YAP, flavonoid

## Abstract

Mesenchymal stem cells (MSCs) are self-renewal and capable of differentiating to various functional cell types, including osteocytes, adipocytes, myoblasts, and chondrocytes. They are, therefore, regarded as a potential source for stem cell therapy. Fisetin is a bioactive flavonoid known as an active antioxidant molecule that has been reported to inhibit cell growth in various cell types. Fisetin was shown to play a role in regulating osteogenic differentiation in animal-derived MSCs; however, its molecular mechanism is not well understood. We, therefore, studied the effect of fisetin on the biological properties of human MSCs derived from chorion tissue and its role in human osteogenesis using MSCs and osteoblast-like cells (SaOs-2) as a model. We found that fisetin inhibited proliferation, migration, and osteogenic differentiation of MSCs as well as human SaOs-2 cells. Fisetin could reduce Yes-associated protein (YAP) activity, which results in downregulation of osteogenic genes and upregulation of fibroblast genes. Further analysis using molecular docking and molecular dynamics simulations suggests that fisetin occupied the hydrophobic TEAD pocket preventing YAP from associating with TEA domain (TEAD). This finding supports the potential application of flavonoids like fisetin as a protein–protein interaction disruptor and also suggesting an implication of fisetin in regulating human osteogenesis.

## 1. Introduction

Fisetin (3,3′,4′,7-tetrahydroxyflavone) is a bioactive flavonoid molecule that has antioxidant, antibacterial, anticancer, and anti-inflammatory cellular activities [1,2,3,4,5]. This molecule can be isolated from many fruits and vegetables, such as strawberry, apple, persimmon, grape, onion, lotus root, kiwi, and cucumber [2,6]. Like other flavonoids, fisetin has two aromatic rings linked via a three-carbon oxygenated heterocyclic ring. It also contains four hydroxyl group substitutions at the 3, 7, 3′, and 4′ positions and one oxo group at the 4 position [1]. Fisetin and other flavonoids have been shown to regulate cell functions, i.e., regulation of vascular smooth muscle contractility [7], protecting against hepatic steatosis [8], also has beneficial effects on anti-neurodegenerative and neuroprotective function [3]. Mechanistically, fisetin could regulate cell function by affecting several important signaling pathways, including PI3K/Akt [9,10], JAK/STAT [11,12], AMPK [8,13], and the Hippo signaling pathway [14]. Furthermore, fisetin can regulate the levels of various growth factors and proinflammatory cytokines, i.e., prostaglandin E2, tumor necrosis factor-alpha (TNF-alpha), interleukin (IL)-1beta, IL-6, and IL-8 depending on the flavonoid structure and the cell types involved [15,16,17]. 

Mesenchymal stem cells (MSCs) are widely regarded as a potential source of stem cell therapy and tissue engineering due to their self-renewal and multilineage differentiation capacity [18,19,20,21,22,23,24,25]. Depending on the culture environment, MSCs are capable of differentiating to various functional cell types, including osteocytes, adipocytes, myoblasts, and chondrocytes [26,27,28]. Several chemical [29,30,31,32], physical [33,34,35,36,37,38], and biological signals [39,40,41,42,43] have been shown to affect MSC functions by activating specific signaling pathways and transcriptional regulators. A previous study reported that fisetin treatment can alter activity of Hippo signaling pathway as fisetin can upregulate expression of leucine zipper containing kinase (ZAK), a kinase in the MAP3K family, to induce the activity of Hippo pathway core-kinases, MST1/2 and LAT1/2, and mediate the activation of JNK/ERK. Activation of these kinases resulted in apoptosis of osteosarcoma cells [14]. We have recently shown that Yes-associated protein (YAP), a transcriptional coactivator that is negatively regulated by Hippo pathway core-kinases, plays a critical role in regulating adipo-osteogenic lineage differentiation of human MSCs. Inhibition of YAP inhibits osteogenic differentiation but promotes adipogenic differentiation of human MSCs [44].

Although several types of flavonoids, such as quercetin, glabridin, and catechin, have been shown to promote the proliferation and osteogenic differentiation of bone marrow-derived mesenchymal stem cells [45,46,47], the effect of fisetin on the biological properties of human MSCs has yet to be studied. In this study, we hypothesized that fisetin plays a role in regulating human osteoblastic differentiation of human MSCs. Accordingly, we set forth to investigate the mechanisms and effects of fisetin on human osteogenic differentiation using two models representing early and late stages of osteoblastic cell differentiation. Human MSCs was used as a representative model for early osteoblastic differentiation, while osteoblast-like SaOs-2 cells were used as a model for studying late stage osteoblasts maturation [48,49]. This finding supports the potential application of flavonoids, such as fisetin, in regulating human osteogenesis.

## 2. Materials and Methods

### 2.1. Isolation and Culture of Human Chorion-Derived Mesenchymal Stem Cells (MSCs)

This study was approved by the ethics committee of the Siriraj Institutional Review Board (SIRB), Faculty of Medicine Siriraj Hospital, Mahidol University, Bangkok, Thailand (COA no. Si112/2020, valid from 7 February 2020 to 6 February 2022).

Human chorionic tissue was collected from placenta of the healthy newborns after receiving written informed consent from their mothers. Placenta was collected by the labor room’s staff and hand-over to our team member for processing. Upon receiving the placenta, the chorionic plate from the region closest to the umbilical cord was dissected, washed three times with Phosphate buffer saline (PBS), cut into small pieces, and treated with 0.25% (*w*/*v*) trypsin-EDTA (GIBCO™; Invitrogen Corporation, Carlsbad, CA, USA) at 37 °C for 30 min. Cells released from the digested tissues were then harvested and washed with PBS, resuspended in Dulbecco’s Modified Eagle Medium (DMEM) supplemented with 10% (*v*/*v*) FBS, and plated into the culture flasks (Corning Incorporated, Corning, NY, USA) [50]. The cells were cultured at 37 °C in a humidified atmosphere containing 5% CO_2_ in air. The culture medium was changed every other day.

### 2.2. Immunophenotypic Characterization

The presence of surface molecules CD73, CD90, and CD105, coupled with the absence of hematopoietic surface molecules CD34 and CD45, is required for the identification of human MSCs [51]. In this study, the derived MSCs were subjected to immunophenotypic characterization as previously described by Lorthongpanich et al. [44]. Briefly, MSCs were washed three times with PBS before incubated with 0.25% (*w*/*v*) trypsin-EDTA (GIBCO™; Invitrogen Corporation, Carlsbad, CA, USA) for 5 min at 37 °C. Suspending cells were harvested and being centrifuged at 1500 rounds per minute (rpm) for 5 min. The supernatant was discarded, and pellet was resuspended in 50 µL of PBS before incubated with 10 µL of fluorescein isothiocyanate (FITC), peridinin-chlorophyll proteins (PerCP) or phyco-erythrin (PE)-conjugated antibodies against CD34, CD45, CD73, CD90, and CD105 (all purchased from BioLegend, San Diego, CA, USA) at 4 °C for 30 min in the dark. After incubation, the cells were washed with PBS and fixed with 1% (*w*/*v*) paraformaldehyde (PFA) before being analyzed by flow cytometer (FACSCantoTM or FACSCaliburTM analyzer; BD Biosciences, San Jose, CA, USA). All antibodies used in this assay were diluted to 1:100 dilutions in PBS.

### 2.3. Culture of the Osteosarcoma Cell Line (SAOS-2)

SaOs-2 cell line was purchased from American Type Culture Collection (ATCC) (Manassas, VA, USA). Cells were cultured in Dulbecco’s Modified Eagle Medium (DMEM) high glucose medium supplemented with 10% fetal calf serum (FCS) and 1% penicillin-streptomycin solution (CORNING, Flintshire, UK) at 37 °C, 5% CO_2_ in air. The cells were subcultured every 4–5 days with 0.25% (*w*/*v*) trypsin-EDTA (GIBCO™; Invitrogen).

### 2.4. Fisetin Preparation

Fisetin was purchased from Sigma-Aldrich Corporation (St. Louis, MO, USA) and was dissolved in dimethyl sulfoxide (DMSO) (Sigma-Aldrich) to obtain a 10 mM stock solution. The stock solution was ultimately diluted in culture medium to obtain the desired concentration. The control group was treated with DMSO at a final concentration of 0.1% to eliminate the effect of the reagent. 

### 2.5. Fisetin Toxicity Test

MSCs or SaOs-2 cells were seeded into 96-well plates (8000 cells/well) and cultured in complete DMEM overnight before being treated with different concentrations of fisetin. At 24 h after treatment, cell viability was determined by the 3-(4,5-dimethylthiazol-2-yl)-2,5-diphenyl-2H-tetrazolium bromide (MTT) assay as described previously [52]. The absorbance at the wavelength of 570 nm was measured by a microplate reader (Synergy H1, BioTek Instruments, Inc., Winooski, VT, USA). The non-cytotoxic concentrations of fisetin that efficiently alter YAP activity were selected for further experiments.

### 2.6. Osteogenic Differentiation and Mineralization Assay

MSCs were seeded into 35 mm tissue culture dishes (Corning Incorporated, Corning, NY, USA) and cultured in DMEM-high glucose supplemented with 10% (*v*/*v*) fetal calf serum (FCS) until their density reach 90% confluence. At this stage, the DMEM was replaced with the NH OsteoDiff® Medium (Miltenyi Biotec, Bergisch Gladbach, Germany) to induce the osteogenic differentiation of human MSCs according to the manufacturer’s instruction. In the fisetin-treated groups, NH OsteoDiff® medium was supplemented with various concentrations of fisetin, range from 0 to 10µM. The medium was replaced every 3 days throughout the entire culture period. Calcium deposition was determined on day 21 by Alizarin Red S staining assay as described previously [53,54]. Briefly, the differentiated MSCs were fixed with 4% (*w*/*v*) PFA for 10 min at 4 °C, washed twice with deionized water, and stained with 40 mM Alizarin Red S (Sigma-Aldrich, USA) for 20 min at room temperature (RT). Calcium deposition of the fisetin-treated SaOs-2 cells was determined using the same protocol, except that the culture duration was shortened to 14 days due to their faster osteogenic differentiation in comparison to the human MSCs.

### 2.7. Alizarin Red S Quantification

The level of calcium deposition was quantified by the amount of Alizarin Red S retained by the cells after staining as previously described [55]. Briefly, 10% (*v*/*v*) acetic acid was added to the Alizarin Red S-stained cells, and the cells were incubated at RT for 30 min with shaking. After incubation, the cells were collected, transferred into a 1.5 mL microcentrifuge tube, and incubated at 85 °C for 10 min. After incubation, the cell mixture was centrifuged at 20,000× *g* for 15 min, the supernatant was collected, and the pH was adjusted to 4.1–4.5 with 10% ammonium hydroxide. Sample aliquots of 50 µL/well were prepared in triplicate in a 96-well plate, and the absorbance at the wavelength of 405 nm was determined by spectrophotometer (BioTek Instruments, Inc., Winooski, VT, USA). The concentration of Alizarin Red S deposited in each sample was calculated using a standard curve generated from various known concentrations of Alizarin Red S.

### 2.8. Alkaline Phosphatase Staining

Cells were cultured in a 35 mm tissue culture dish with and without fisetin supplementation for 21 days. The cells were then stained with an alkaline phosphatase staining kit according to the manufacturer’s protocol (SK-5100; Vector Laboratories, Burlingame, CA, USA). 

### 2.9. Wound Healing Assay

MSCs (passage 3–6) or SaOs-2 cells were seeded at a density of 5 × 10^5^ cells/cm^2^ in a Culture-Insert 2 well in µ-dish 35 mm (ibidi, GmbH, Gräfelfing, Germany), and the cells were allowed to settle. Once the cells attached to the dish, the Culture-Insert was removed, and the dish was filled with medium supplemented with different concentrations of fisetin. The dynamic activity of the cells was recorded by PAULA® time-lapse microscopy (Leica Microsystems, Wetzlar, Germany) [56]. Images of the closing wound were automatically acquired every 10 min. The migration rate and the time until 50% and 100% gap closure were recorded. The migration index was calculated using the following equation: migration index = 1 − ((the width of the scratch at 0 hours [Width0h] − the width of the scratch at T h [Width T h])/Width 0 h) [57,58].

### 2.10. Transwell Migration Assay

The MSCs or SaOs-2 cells were treated with 0, 1, 10, or 30 µM fisetin for 24 h before being seeded into the insert chamber of an 8 μm pore size transwell (Corning) filled with DMEM supplemented with 2% (*v*/*v*) fetal bovine serum (FBS), 100 U/mL penicillin, and 100 μg/mL streptomycin. The lower chamber contained 600 μL of DMEM medium supplemented with 20% FBS. The culture was then maintained for 6 or 48 h to allow cell migration at 37 °C, 5% CO_2_ in air. Cells that migrated through the transwell chamber were fixed with 4% (*x*/*v*) paraformaldehyde for 20 mins, washed with PBS before staining with Hoechst 33,342 (Thermo Scientific, MA, USA), and subjected to cell count analysis. Three independent experiments were performed. Data are presented as the mean ± standard deviation (SD). Evaluation of the migration of SaOs-2 cells was performed using the same protocol [59].

### 2.11. Real-Time Quantitative Reverse Transcription Polymerase Chain Reaction (Real-Time qRT-PCR)

Total RNA was isolated and reverse-transcribed using a High-Capacity cDNA Reverse Transcription Kit (Applied Biosystems, Foster City, CA, USA). Real-time qRT-PCR was performed using Real-Time PCR Master Mix (Applied Biosystems). Real-time qRT-PCR assays were performed using a CFX384 Real-Time PCR System (Bio-Rad Laboratories, Hercules, CA, USA). RNA isolation, reverse-transcription, and qRT-PCR were conducted according to the manufacturer’s instruction. The primers used in this study are listed in Supplementary Table S1.

### 2.12. Western Blot Analysis

The presence of Collagen type I and YAP was determined by Western blotting as previously described by Lorthongpanich et al., [44]. Briefly, the protein was isolated from the differentiated cells using a cell lysis buffer (10x (radioimmunoprecipitation assay) RIPA buffer; Cell Signaling Technology, Danvers, MA, USA) containing protease inhibitors (Roche Life Science, Penzberg, Germany). The denatured protein was loaded onto 7% or 12% sodium dodecyl sulfate (SDS)/polyacrylamide gels, and the separated proteins were transferred to polyvinylidene difluoride (PVDF) membranes (Merck Millipore, Burlington, MA, USA) and probed with the following primary antibodies: anti-collagen type I, anti-YAP (Cell Signaling Technology) diluted 1:1000, and anti-β-actin-peroxidase (ACTB; Sigma-Aldrich) diluted 1:25,000. Peroxidase-conjugated, species-appropriate antibody at a 1:5000 dilution was added. The signal was detected by autoradiography using enhanced chemoluminescence (Merck Millipore). ACTB served as the loading control. 

### 2.13. Computational Method

The crystal structure of human YAP and TEA domain (TEAD) complex (PDB ID: 3KYS) was obtained from the Protein Data Bank [60], and the structure of fisetin was obtained from the PubChem database [61]. All amino acids were protonated at pH 7.4 using H^++^ server [62]. Gaussian09 program [63] was employed using the HF/6-31G* basis set to calculate the electrostatic potential (ESP) charges of fisetin. The restrained ESP (RESP) charges of fisetin were obtained using the antechamber modules of AMBER20 [64]. Other parameters were generated using General AMBER Force Field Version 2 (GAFF2) [65]. Remaining missing parameters were obtained using parmchk2 modules [66]. Autodock Vina was employed to predict the binding conformation of fisetin on TEAD [67]. LEaP module in AMBER20 was used to solvate TEAD–YAP complex and TEAD–YAP–fisetin complex in an isomeric truncated octahedral TIP3P water box with the buffer distance of 13 Å. Sodium ions (Na^+^) were added to neutralize the systems [68]. To remove unfavorable interactions in the systems, minimization procedure was performed with 2500 steps of steepest-descent followed by 2500 steps of conjugated gradient in each step. First, the heavy atoms of proteins were restrained with force constant of 5.0 kcal/(mol Å2). The protein backbones were then restrained with force constants of 10, 5, and 1 kcal/(mol Å2), respectively. Lastly, the systems were freely minimized. Next, PMEMD module (AMBER) was employed with the SHAKE algorithm (AMBER) to simulate the systems under the periodic boundary condition while constraining all bonds involving hydrogen atoms with a simulation timestep of 0.002 ps [69]. Temperatures were controlled using Langevin dynamics technique [70] with a collision frequency of 1 ps−1. Each system was heated from 0 K to 310 K for 200 ps while the protein backbones were restrained with force constant of 10 kcal/(mol Å2) in the NVT ensemble. The systems were simulated at 310 K for 300 ps in the NVT ensemble. After that, the systems were simulated at 310 K and 1 atm in the NPT ensemble for 40 ns. The root-mean-square deviation (RMSD) value of each simulated structure in each system was computed with respect to the minimized structure, and these values were used to analyze their stability. MMPBSA.py module (AMBER) [71] was used to evaluate the binding affinity between TEAD and YAD of both complexes by calculating the total binding free energy (${\Delta G}_{\mathrm{bind}}$). This module was also used to determine the key binding residues of TEAD involved in fisetin binding of the TEAD–YAP–fisetin complex by calculating per-residue decomposition free energy (${\Delta G}_{\mathrm{bind}}^{\mathrm{residue}}$). These calculations were performed on the last 10 ns trajectories based on molecular mechanics/Poisson–Boltzmann (generalized Born) surface area (MM/PB(GB)SA) methods [72].

### 2.14. Statistical Analysis

The results are presented as mean ± standard deviation (SD). Mann–Whitney U test was used to compare non-parametric variations between groups. A *p*-value of <0.05 was considered to be statistically significant. The data were analyzed by GraphPad Prism software version 8.0 for Windows (GraphPad Software, La Jolla, CA, USA). 

## 3. Results and Discussion

### 3.1. Fisetin Suppresses MSC Proliferation and Migration in a Dose-Dependent Manner

**Figure 1:**
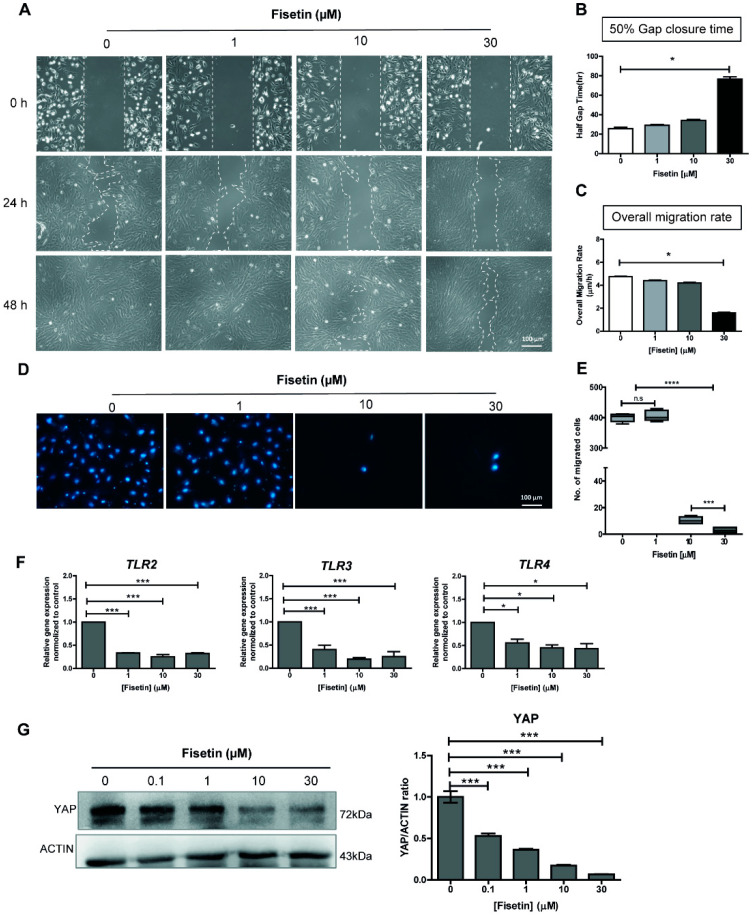
Effect of fisetin on proliferation and migration of MSCs. A scratch was made using a P1000 pipette tip and cultured in the presence of different concentrations of fisetin for 48 h (**A**). The distance between edges was monitored by PAULA^®^ time-lapse microscopy to determine the 50% gap closure time (**B**), and the overall migration rate upon fisetin treatment (**C**). Representative pictures of cells at the bottom side of the inserted chamber after 6 h incubation stained with Hoechst-33342 (**D**). The number of migrated cells was counted and reported as mean ± SD; *n* = 3 (**E**). Expression level of TLR2, TLR3, and TLR4 genes upon fisetin treatment (**F**). YAP expression upon fisetin treatment (as determined by Western blotting) and quantitative data (**G**). The data presented in subfigure (**B**–**E**) were analyzed Mann–Whitney U test. Bars indicate mean values + standard deviation (SD) and asterisks indicate differences * *p* < 0.05; *** *p* < 0.001; **** *p* < 0.0001, n.s. = no significant difference.

Human chorionic tissue-derived MSCs characterized by immunophenotypic profiling using flow cytometry was used in this study (Supplementary Figure S1). The effects of fisetin on MSC proliferation and migration were determined by wound healing and transwell migration assays. The results from wound healing assay showed that fisetin, at doses above 10 µM, inhibited wound closure and significantly reduced overall MSC migration in a dose-dependent manner (Figure 1A–C). In agreement with the wound healing assay, fisetin, at 10 µM and 30 µM, also reduced MSC migration in the transwell migration assay by approximately 40-fold. As shown in Figure 1D,E, the number of migratory MSCs in the group treated with 1µM fisetin was not changed when compared with control (404.9 ± 15 cells vs. 399.5 ± 12 cells). However, a significant reduction of migratory cells was observed in groups treated with 10 µM and 30 µM fisetin (10.4 ± 2.1 cells and 2.9 ± 1.6 cells, respectively). Taken together, these results clearly show that fisetin, at concentrations above 10 µM, suppressed MSC proliferation and migration. However, the inhibitory effects of fisetin on the migration and invasion of various cancer cell types have been previously demonstrated [4,10]. This is, to the best of our knowledge, the first study which demonstrates the inhibitory effects of fisetin on the proliferation and migration of human chorionic tissue-derived MSCs.

### 3.2. Fisetin Inhibits the Expressions of Toll-Like Receptors and YAP Protein

To understand the mechanisms underlying the negative effects of fisetin on MSC proliferation and migration, we investigated the effects of fisetin on the expression levels of Yes-associated protein 1 (YAP) and several Toll-like receptors (TLRs). It has been shown that TLR signaling regulates the proliferation, migration, and differentiation of many cell types, including MSCs [73], and that one of its targets (YAP) plays an important role in the regulation of human MSC proliferation and migration [74,75]. Although a previous study reported that fisetin inhibits TLR4 expression in lung tissue [76], the effect of fisetin on TLR signaling and YAP expression in MSCs has yet to be characterized. As shown in Figure 1F, 1 µM fisetin significantly downregulated the expression levels of TLR2, TLR3, and TLR4 genes. Further increasing fisetin concentrations did not show an additional suppressive effect on the expression of those TLR genes. Further investigation found that fisetin reduced the expression level of YAP protein in a dose-dependent manner as determined by Western blotting (Figure 1G). The downregulation of these TLRs and the YAP protein as a result of fisetin treatment may compromise the ability of MSCs to proliferate and migrate. 

### 3.3. MSCs Failed to Undergo Osteogenic Differentiation upon Fisetin Treatment

**Figure 2:**
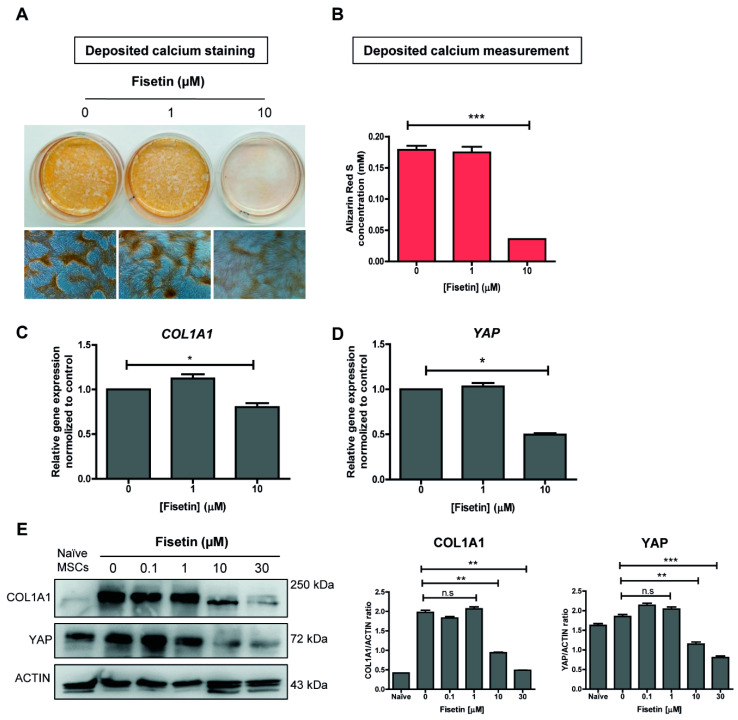
Osteogenic differentiation of MSCs upon fisetin treatment. Fisetin treatment at 10 µM inhibits osteogenic differentiation (as determined by Alizarin Red S staining) and quantitative measurement (**A**,**B**). Transcriptional analysis of COL1A1 and YAP (**C**,**D**) and protein expression, and the ratio of COL1A1 and YAP to ACTIN (**E**) upon fisetin treatment as determined by quantitative RT-PCR and western blotting, respectively. The data presented in subfigure (**B**–**E**) were analyzed by Mann–Whitney U test. Bars indicate mean values ± standard deviation (SD) and asterisks indicate significant differences, * *p* < 0.05; ** *p* < 0.01; *** *p* < 0.001, n.s. = no significant difference.

In addition to the effect of YAP on MSC proliferation and migration, a reduction of YAP has also been shown to inhibit osteogenic differentiation of MSCs [44]. We, therefore, set forth to determine the effect of fisetin on osteogenic differentiation of MSCs using an in vitro culture system. After 21 days of differentiation, calcium deposition in the differentiated MSCs was determined by Alizarin Red S staining (Figure 2A,B). The result showed that 10 µM fisetin suppressed osteogenic differentiation of human MSCs as demonstrated by the significant reduction in calcium deposition at the end of their osteogenic differentiation. Moreover, 10 µM fisetin also significantly down-regulated the expression of YAP and collagen type I alpha 1 chain (COL1A1), a well-known osteoblastic marker, at both mRNA (Figure 2C,D) and proteins levels (Figure 2E). Taken together, these results suggest that 10 µM fisetin inhibited the osteogenic differentiation of human MSCs, possibly by down-regulating YAP expression. Our result is contradicted by a previous report in mouse which shows that fisetin promoted osteoblastic differentiation in vivo [77]. The difference might arise from the cell types (mouse vs. human) and the different osteogenic differentiation method used in each study (in vivo vs. in vitro).

### 3.4. Fisetin Inhibits the Proliferation, Migration, and Maturation of Osteoblast-Like Cells

**Figure 3:**
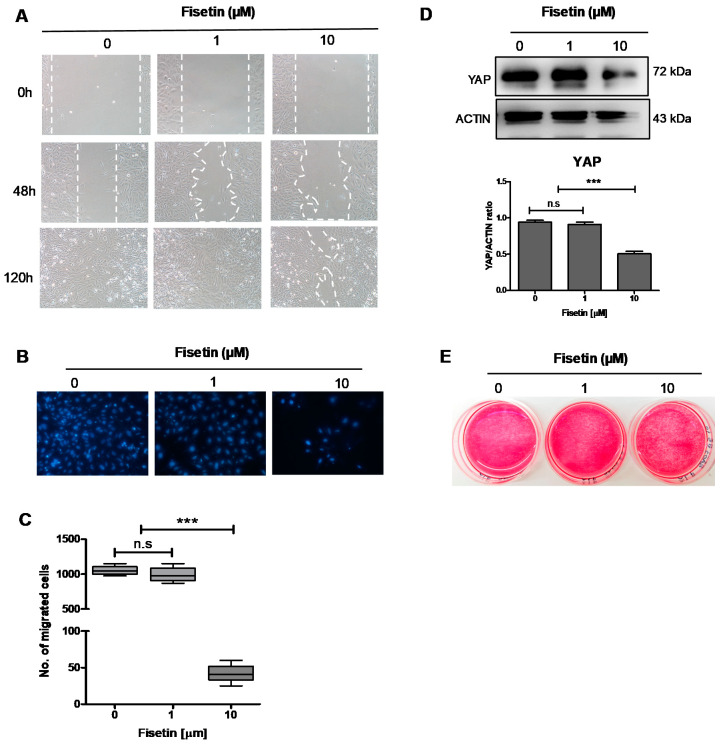
Effect of fisetin on proliferation and migration of SaOs-2 cells. The distance between edges was monitored under inverted microscope to determine gap closure upon fisetin treatment (**A**). Representative pictures of cells at the bottom side of the inserted chamber after 48 h incubation stained with Hoechst-33342 (**B**). The number of migrated cells was counted and reported as mean ± SD; *n* = 3 (**C**). YAP expression in SaOs-2 cells upon fisetin treatment for 48 h (**D**). Alkaline phosphatase activity staining (**E**). The data presented in subfigure (**C**,**D**) were analyzed by Mann–Whitney U test. Bars indicate mean values ± standard deviation (SD) and asterisks indicate significant differences, *** *p* < 0.001; n.s. = no significant difference.

Osteogenic differentiation is a multistep process in which the MSCs initially differentiate to proliferative osteoblasts that are gradually matured to become osteocytes embedded in the mineralized extracellular matrix [78,79]. To study the role of fisetin during the osteoblastic proliferation and maturation process, human osteosarcoma SaOs-2 cells, which are widely used as a model for studying osteoblastic cell growth and maturation [48,49], were subjected to fisetin treatment. Similar to its effects on MSCs, 10 µM fisetin significantly inhibited SaOs-2 cell proliferation and migration as determined by both wound healing and transwell migration assays (Figure 3A–C). Moreover, 10 µM fisetin also reduced YAP expression in SaOs-2 cells in a manner similar to that observed in MSCs (Figure 3D). These results suggest that fisetin inhibits the proliferation and migration of osteoblasts. However, fisetin treatment did not significantly inhibit alkaline phosphatase activity in SaOs-2 cells (Figure 3E).

**Figure 4:**
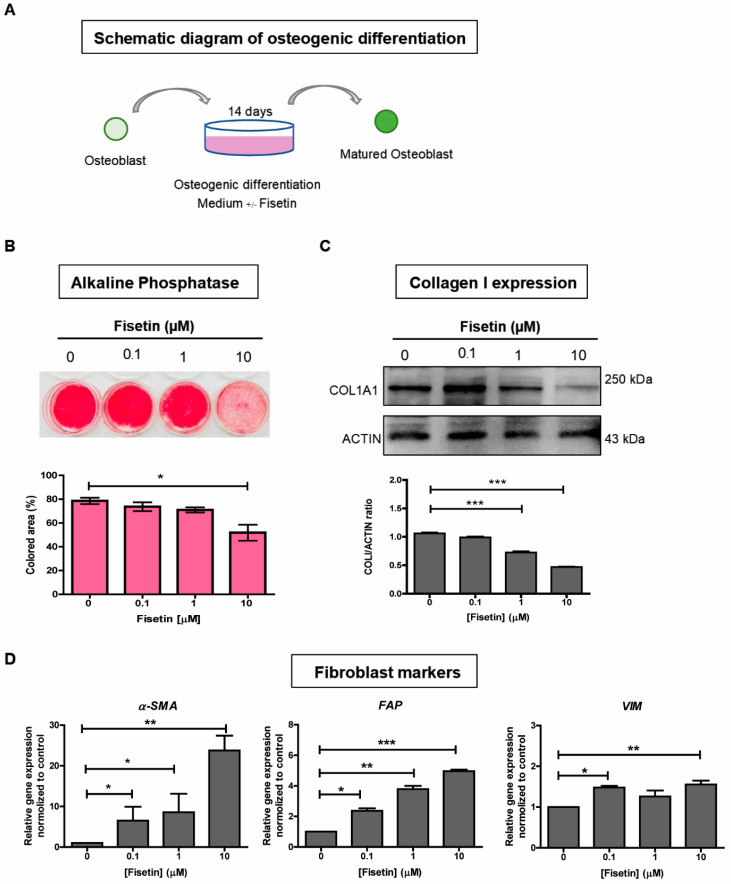
Fisetin inhibits osteogenic differentiation of SaOs-2 cells. Schematic diagram of osteogenic differentiation to induce osteoblasts to become mature osteoblasts (**A**). Alkaline phosphatase staining and colored area measurement (**B**). Expression of the COL1A1 protein as determined by Western blot analysis, and the ratio of COL1A1 to ACTIN (**C**). Quantitative analysis for the transcriptional expression of mesenchyme and fibroblast markers after SaOs-2 cells were treated with fisetin (**D**). The data presented in subfigure (**B**–**D**) were analyzed by Mann–Whitney U test. Bars indicate mean values ± standard deviation (SD) and asterisks indicate significant differences, * *p* < 0.05; ** *p* < 0.01; *** *p* < 0.001.

We next investigated the effect of fisetin on the maturation of osteoblastic cells by culturing SaOs-2 cells in osteogenic differentiation medium in the presence of fisetin (Figure 4A). After 14 days of culture, a significant reduction in the expression levels of the osteogenic marker alkaline phosphatase and COL1A1 were observed in 10 µM fisetin-treated SaOs-2 cells (Figure 4B,C). In contrast to the reduced expression observed in osteogenic markers, fisetin significantly upregulated the expression levels of several fibroblast marker genes, including α-smooth muscle actin (α-SMA), fibroblast activation protein (FAP), and vimentin (VIM) in SaOs-2 cells (Figure 4D). These results suggest that fisetin inhibits the maturation of osteoblasts by inhibiting the expression levels of osteogenic genes and dedifferentiates those cells toward more primitive fibroblasts by enhancing the expression levels of fibroblast genes. Taken together, these results suggest that fisetin inhibits the proliferation, migration, and maturation of osteoblasts, possibly via the inhibition of YAP expression.

### 3.5. Targeting YAP in Osteoblast-Like Cells Recapitulates Fisetin-Induced Phenotypes

**Figure 5:**
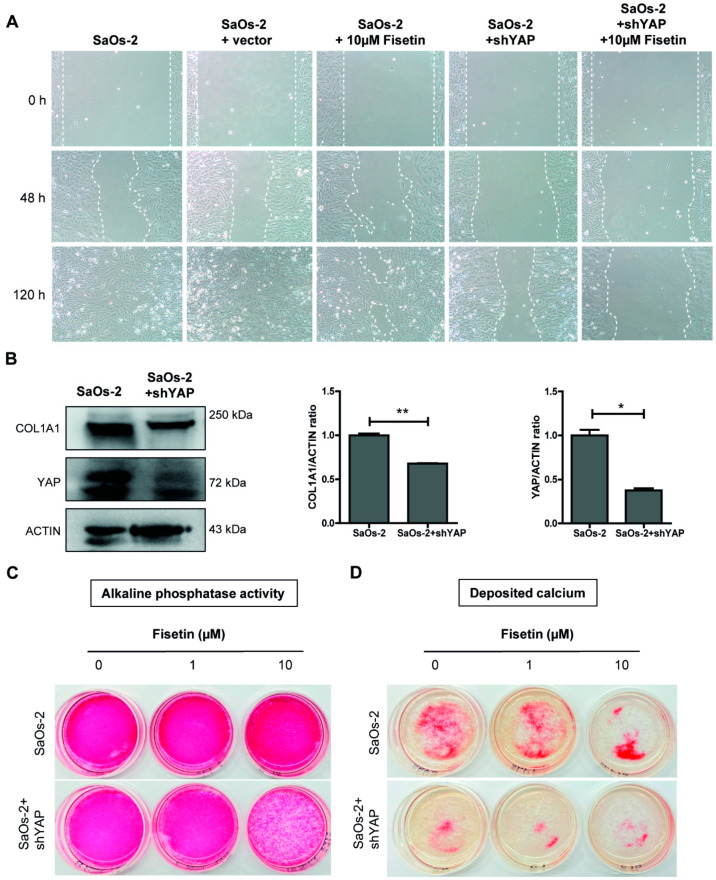
Targeting YAP recapitulates fisetin treatment phenotype. Control SaOs-2 cells and SaOs-2-YAP-KD cells were treated with fisetin during wound healing assay (**A**). Expression of COL1A1 and YAP, and the ratio of COL1A1 and YAP to ACTIN in SaOs-2 and SaOs-2+shYAP cells at 14 days after culturing in osteogenic differentiation medium (**B**). SaOs-2 and SaOs-2+shYAP were cultured in osteogenic differentiation medium with or without fisetin and subjected to alkaline phosphatase staining (**C**) and mineralization assay (**D**). The data presented in subfigure (**B**) were analyzed by Mann–Whitney U test. Bars indicate mean values ± standard deviation (SD) and asterisks indicate significant differences, * *p* < 0.05; ** *p* < 0.01.

To further investigate the role of YAP in fisetin-mediated effects on the proliferation, migration, and maturation of osteoblasts, a knockdown experiment using shRNA targeting YAP was performed in SaOs-2 cells. Western blot analysis showed 40% and 65% reduction of both COL1A1 and YAP proteins, compared with control (Figure 5B). The SaOs-2-YAP-KD (SaOs-2 + shYAP) cells that expressed lower levels of YAP were then subjected to wound healing and osteogenic differentiation assays. The results showed that the reduction of YAP protein via the knockdown of YAP expression inhibited the proliferation and migration of SaOs-2 cells in a similar manner to that of SaOs-2 cells treated with 10 µM fisetin. Moreover, the addition of 10 µM fisetin further enhanced the effects of YAP-KD to a level greater than that observed in either treatment alone (Figure 5A and Supplementary Figure S2). 

We next determined the role of YAP in the maturation of osteoblasts by inducing the osteogenic differentiation of SaOs-2-YAP-KD cells. A reduction of the osteogenic markers COL1A1 and alkaline phosphatase was observed in SaOs-2-YAP-KD cells (Figure 5B,C). The level of matrix mineralization in the differentiated SaOs-2-YAP-KD cells as determined by Alizarin Red S staining was also reduced (Figure 5D). Moreover, the addition of 10 µM fisetin further enhanced the negative effects of YAP-KD on the osteogenic differentiation and matrix mineralization of SaOs-2-YAP-KD cells (Figure 5D). A reduction of Alizarin Red S staining was also observed in MSC-YAP-KD cells (Supplementary Figure S3). These results suggest that 10 µM fisetin inhibits the proliferation, migration, and maturation of human osteoblast-like cells, possibly by downregulating the expression level of YAP. The synergistic effect of fisetin on YAP-KD is possibly derived from the additional inhibitory effect of fisetin on the already lower level of YAP in SaOs-2-YAP-KD cells.

### 3.6. Molecular Docking and Molecular Dynamic Simulation

**Figure 6:**
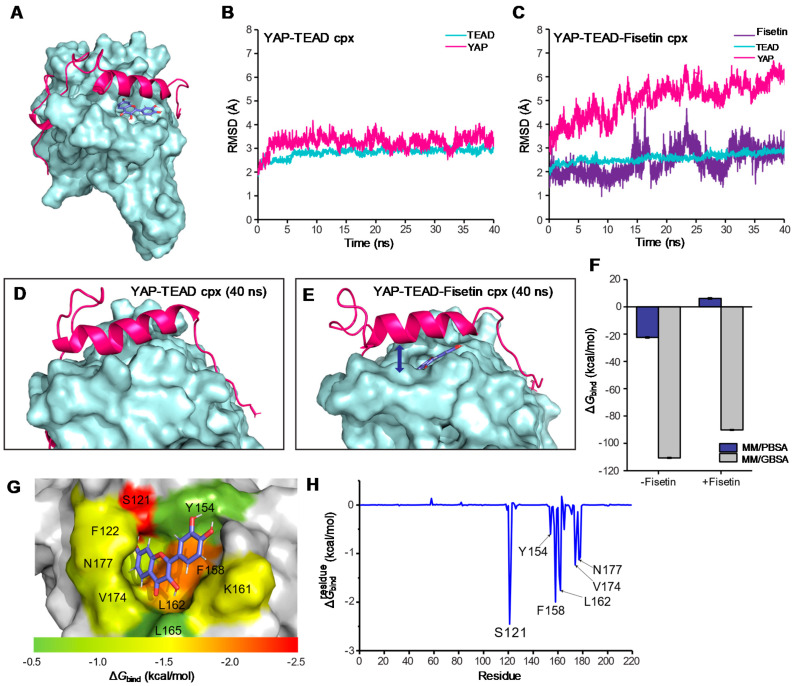
The binding conformation and binding energy of fisetin to the YAP–TEAD complex as predicted by molecular docking and molecular dynamic simulation. (**A**) Overall binding conformation of fisetin to the YAP–TEAD complex. (**B**) All atom RMSD of YAP and TEAD in apo form. (**C**) All atom RMSD of YAP and TEAD in fisetin-bound form. (**D**) The apo form of the YAP–TEAD binding interface at 40 ns trajectory of MD. (**E**) The fisetin-bound form of the YAP–TEAD binding interface at 40 ns trajectory of MD. (**F**) YAP–TEAD binding free energy (∆Gbind) with and without fisetin. (**G**–**H**) ∆$G_{\mathrm{bind}}^{\mathrm{residue}}$ values of fisetin in complex with TEAD. The important residues involved in fisetin binding are colored according to their ∆$G_{\mathrm{bind}}^{\mathrm{residue}}$ values.

To further elucidate how fisetin could reduce YAP activity, we hypothesized that fisetin might disrupt YAP–TEAD protein–protein interaction and inhibit YAP–TEAD activity. To prove this hypothesis, molecular docking and molecular dynamics (MD) simulations were performed. Fisetin was blindly docked to the TEAD protein (PDB: 3KYS), and the conformer that bound at the YAP–TEAD interface was selected for simulation (Figure 6A). Based on the RMSD value, YAP and TEAD in the YAP–TEAD complex were found to be stable around 3.4 Å since approximately 3 ns of simulation (Figure 6B). In contrast, the RMSD values of YAP in the YAP–TEAD–fisetin complex were gradually increased for all 40 ns of simulation and reached around 5.4 Å at the end (Figure 6C). This indicates that YAP could not stably bind to TEAD when fisetin was presented on the interface of the complex. The structure of the YAP–TEAD complex at a 40 ns trajectory clearly showed that the gap between YAP and TEAD is widened when fisetin bound to their interface (Figure 6D,E). The binding free energy (∆Gbind) of YAP to TEAD was calculated from the last 10 ns trajectories of simulation. As shown in Figure 6F, the ∆Gbind of YAP in the YAP–TEAD–fisetin complex (∆$G_{\mathrm{bind}}^{\mathrm{MM}/\mathrm{GBSA}}$ = −90.18 ± 0.29 kcal/mol; ∆$G_{\mathrm{bind}}^{\mathrm{MM}/\mathrm{PBSA}}$ = 6.14 ± 0.51 kcal/mol) was significantly higher than in those of the YAP–TEAD complex (∆$G_{\mathrm{bind}}^{\mathrm{MM}/\mathrm{GBSA}}$ = −110.65 ± 0.34 kcal/mol; ∆$G_{\mathrm{bind}}^{\mathrm{MM}/\mathrm{PBSA}}$ = −22.46 ± 0.42 kcal/mol). These results suggest that the binding of fisetin to the YAP–TEAD complex could affect the binding conformation and reduced the affinity of YAP to TEAD. 

Further investigation on the binding conformation of fisetin to TEAD found that the planar flavonol ring of fisetin occupied the hydrophobic TEAD pocket composed of F122, Y154, F158, K161, L162, V174, L175, N177, and F178, and formed polar contact with S121 which positions near interface 2 of TEAD (Figure 6G,H). The predicted inhibition mechanism of fisetin also resembled CPD3.1, that the compound could bind to the YAP–TEAD interaction interface and inhibit TEAD activity [80]. Binding of small molecules to the YAP–TEAD interface might also affect protein stability [81]. As demonstrated here that fisetin occupied the hydrophobic TEAD pocket; therefore, it suggests fisetin as a protein–protein interaction disruptor that disrupts the YAP–TEAD interaction and also suggesting an implication of fisetin for their therapeutic application.

## 4. Conclusions

Flavonoid fisetin inhibited the proliferation, migration, and osteogenic differentiation of MSCs and SaOs-2 cells by reducing YAP activity, which resulted in the downregulation of osteogenic genes and the upregulation of fibroblast genes. Further analysis using molecular docking and molecular dynamics (MD) simulations suggests that fisetin disrupts YAP activity by preventing it from associating with its partner TEAD transcription factor. Altogether, our results suggest that fisetin might inhibit the activity of Hippo signaling pathway by disrupting the interaction between YAP and TEAD proteins and could potentially be used to modulate Hippo signaling pathway in the future therapeutic application.

## Data Availability

Data is contained within the article or Supplementary Materials.

## Supplementary Materials

The following are available online at https://www.mdpi.com/article/10.3390/antiox10060879/s1.

## Abbreviations

MSCs	Mesenchymal stem cells
YAP	Yes-associated protein
YAP-KD	YAP knock-down
TLRs	Toll-like receptors
shRNA	Short hairpin RNA
PBS	Phosphate buffer saline
DMEM	Dulbecco’s Modified Eagle Medium
FCS	Fetal Calf Serum
DMSO	Dimethyl sulfoxide
PVDF	Polyvinylidene difluoride
COL1A1	Collagen type 1
α-SMA	α-smooth muscle actin
FAP	Fibroblast activation protein
VIM	Vimentin
