# Supplementary figures and images for: Fisetin Inhibits Osteogenic Differentiation of Mesenchymal Stem Cells via the Inhibition of YAP
Source: Antioxidants (Basel). 2021 May 30;10(6):879. doi: 10.3390/antiox10060879 (PMC8226865; doi:10.3390/antiox10060879)

Lorthongpanich\_Sup\_Fig 1

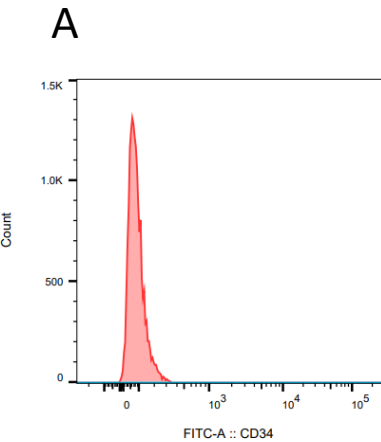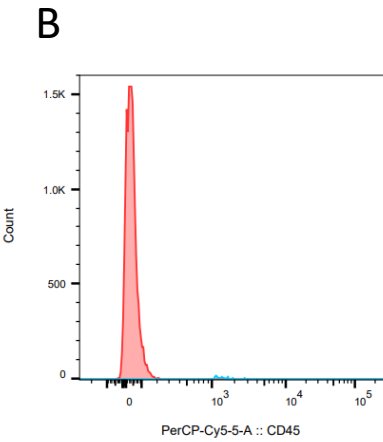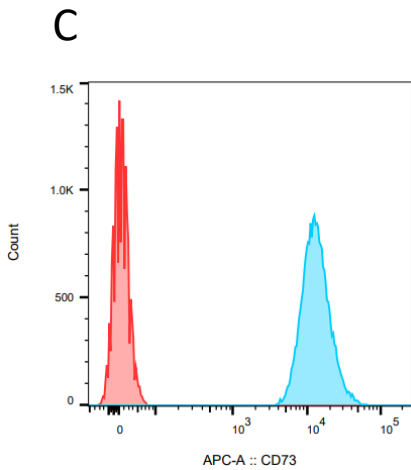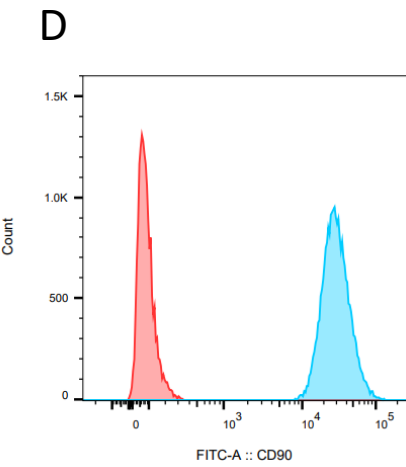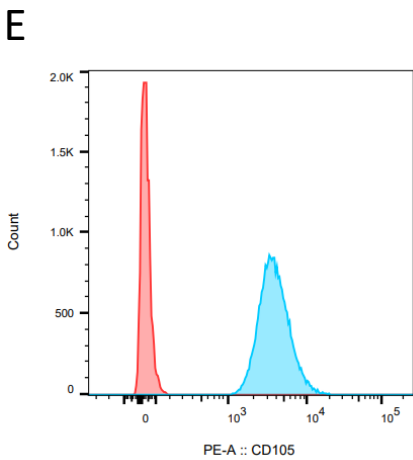

Lorthongpanich\_Sup\_Fig 2

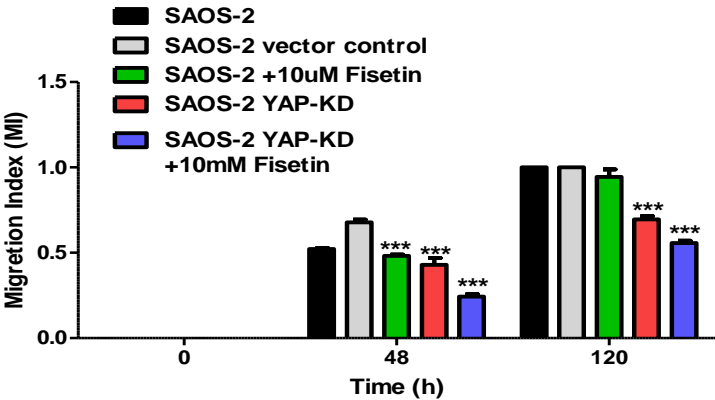

Lorthongpanich\_Sup\_Fig 3

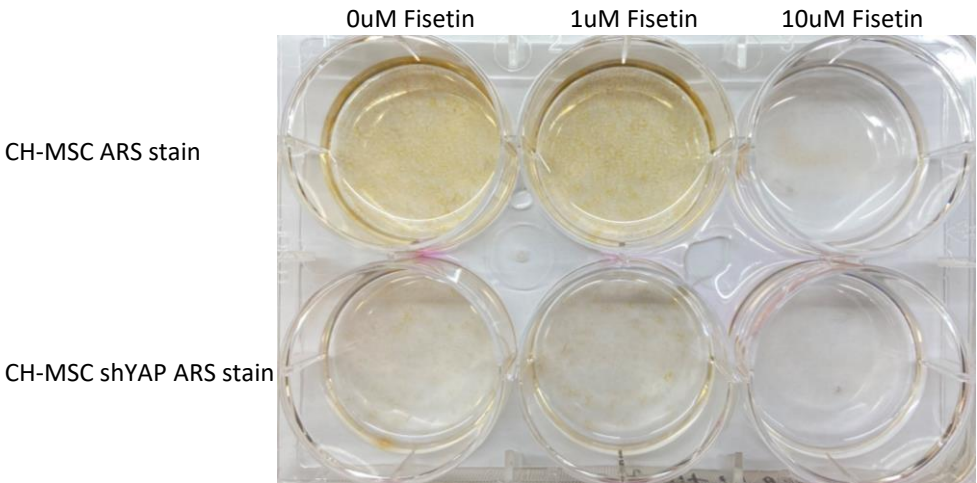

Supplement: Supplementary file 1 [file antioxidants-10-00879-s001.zip › antioxidants-1214031-supplementary.pdf]
